# The effects of amplitude modulated transcranial alternating current stimulation on working memory of college students

**DOI:** 10.3389/fnhum.2025.1639378

**Published:** 2025-10-20

**Authors:** Heng Wang, Yan Chen, Ziyu Zhou, Rui Jiang, Haowei Hu, Yan Zhao, K. Dimyati, Shen Tong, Ji Wang, Xiao Zhang

**Affiliations:** ^1^School of Medical Information and Engineering, Xuzhou Medical University, Xuzhou, China; ^2^Information Center, Chengdu Second People’s Hospital, Chengdu, China; ^3^Xuzhou Jiazhi Technology Co., LTD, Xuzhou, China; ^4^Yantai Longchi Technologies Co., LTD, Yantai, China; ^5^Department of Electrical Engineering, Faculty of Engineering, Centre of Advanced Communication Research and Innovation, Universiti Malaya, Kuala Lumpur, Malaysia; ^6^Department of Children Healthcare, Xuzhou Children’s Hospital, Xuzhou, China; ^7^School of Medicine and Health, Harbin Institute of Technology, Harbin, China

**Keywords:** amplitude modulated transcranial alternating current stimulation (AM-tACS), working memory (WM), electroencephalography (EEG), brain stimulation, transcranial alternating current stimulation (tACS)

## Abstract

**Background:**

Recent studies suggest that amplitude-modulated transcranial alternating current stimulation (AM-tACS) may enhance cognitive functions, but its mechanisms and optimal application remain unclear.

**Methods:**

Thirty-three healthy university students were randomly assigned to Sham, tACS (40 Hz, 1 mA, bilateral prefrontal cortex), or AM-tACS (200 Hz carrier frequency) groups, in AM-tACS, the baseband modulation frequency was individualized based on the pre-task phase-locking value (PLV) derived from occipitofrontal EEG. Working memory (WM) was assessed via a delayed-match-to-sample task (accuracy and sensitivity index d’).

**Results:**

Compared to Sham, the tACS group showed significant WM accuracy improvement (*p* < 0.05). AM-tACS exhibited a smaller but statistically significant enhancement in d’ (*p* < 0.05). EEG analysis revealed no PLV increase between stimulated regions, but a trend toward heightened frontal-occipital functional connectivity.

**Conclusion:**

Amplitude-modulated transcranial alternating current stimulation effectively enhances WM in college students, though physiological mechanisms require further investigation with multimodal approaches. The compatibility of AM-tACS with real-time EEG monitoring highlights its potential for closed-loop neuromodulation systems, where stimulation parameters could be dynamically adjusted based on neural feedback.

## 1 Introduction

Working memory (WM) constitutes a fundamental cognitive system that enables the temporary storage and manipulation of information essential for complex thought processes. As a core component of executive functioning, WM capacity not only predicts academic and professional achievement but also serves as a sensitive marker of cognitive health (Baddeley, 2012; Borghini et al., 2018; Ziaei et al., 2017). The clinical relevance of WM is underscored by its impairment across multiple neuropsychiatric disorders, including schizophrenia, attention-deficit/hyperactivity disorder (ADHD), and neurodegenerative conditions (Dunning et al., 2016; Grot et al., 2017; Le et al., 2017; Maehler and Schuchardt, 2016; Phillips et al., 2017; Stegmayer et al., 2015). These robust associations position WM as both a diagnostic indicator and therapeutic target for cognitive enhancement interventions.

Among available neuromodulation approaches, transcranial alternating current stimulation (tACS) has emerged as particularly promising for WM enhancement due to its unique combination of temporal precision and frequency specificity (Benussi et al., 2022; Booth et al., 2022; Grover et al., 2022; Johnson et al., 2023; Nissim et al., 2023; Wu et al., 2021). By applying weak alternating currents tuned to gamma-band frequencies (40 Hz), which reflect the natural rhythmicity of WM-related neural activity, tACS can selectively entrain oscillations in prefrontal cortical networks (Wischnewski et al., 2023; Asamoah et al., 2022). However, conventional tACS faces three major limitations: (1) interindividual variability in optimal stimulation frequencies, (2) limited spatial specificity due to current dispersion, and (3) fixed amplitudes that fail to capture dynamic physiological modulation during WM tasks.

To address these limitations, we introduce amplitude-modulated tACS (AM-tACS) with two innovative features: (1) a 200 Hz carrier frequency with individualized baseband modulation frequency (determined by pre-task phase-locking value from occipitofrontal EEG) to better mimic natural cortical oscillations (Kasten et al., 2018; Hsu et al., 2023; Thiele et al., 2021; Haslacher et al., 2021), and (2) individualized frequency selection while maintaining standard 1 mA peak-to-peak current intensity for safety. This design builds on established findings that successful WM performance involves characteristic theta-range (4–8 Hz) amplitude fluctuations in neural synchronization (Haslacher et al., 2023; Omae et al., 2024).

To comprehensively evaluate AM-tACS effects, we employ high-density electroencephalography (EEG) for three critical capabilities: (1) millisecond temporal resolution to track stimulation-induced oscillatory dynamics, (2) spectral power analysis to complement behavioral measures, and (3) phase-based connectivity metrics (e.g., phase-locking value) to investigate network-level mechanisms. This multimodal approach enables testing our central hypothesis that AM-tACS enhances WM performance by optimizing theta-gamma cross-frequency coupling between frontal executive and posterior storage systems.

The potential implications of this work span both basic and translational neuroscience. By establishing AM-tACS efficacy and mechanisms in healthy adults, we lay the groundwork for developing closed-loop neuromodulation systems that adapt stimulation parameters via real-time neural feedback. Such advances could transform treatment for WM impairments across neuropsychiatric disorders while informing next-generation neurotechnologies that combine surgical-level precision with non-invasive practicality.

## 2 Materials and methods

### 2.1 Participants

This study was approved by the Medical Ethics Committee of Xuzhou Medical University Affiliated Hospital (Ethics No. XYFY2023-KL399-01) and was conducted in strict accordance with the Declaration of Helsinki. The development of effective WM interventions requires careful consideration of both target population and intervention methodology. In this study, we focus on healthy university students–a population that offers three key advantages for establishing foundational neuromodulation parameters: (1) neurodevelopmental stability minimizing age-related variability in cognitive processing, (2) consistent task engagement ensuring reliable measurement of intervention effects, and (3) demographic homogeneity facilitating clinical translation to patient populations with WM impairments.

In total, 35 participants were recruited from within Xuzhou Medical University. All participants signed written informed consent forms after fully understanding the study procedures and potential risks, and were compensated appropriately. All participants were randomly assigned to one of three experimental groups: Sham (*n* = 11), tACS (*n* = 13), or AM-tACS (*n* = 11). During the experiment, one participant in the tACS group withdrew due to discomfort caused by electrical stimulation, and another in the Sham group was excluded for excessive drowsiness. Consequently, the final sample sizes were Sham = 10, tACS = 12, and AM-tACS = 11. Thus, data of 33 participants (14 male) between the age of 18 and 32 (mean 22.1 ± 3.1 years) was evaluated as part of this work.

### 2.2 Experimental design

**FIGURE 1:**
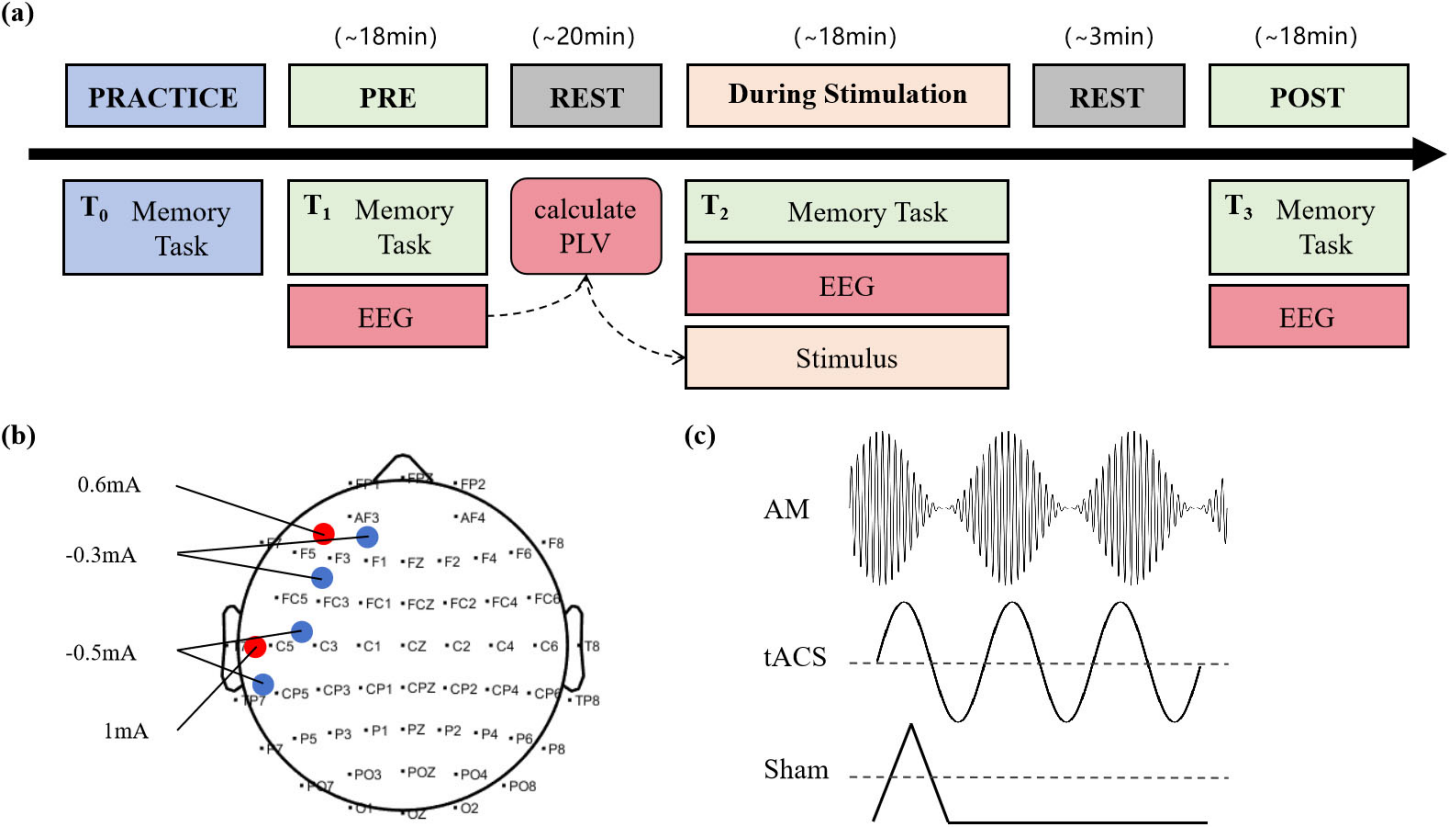
Experimental procedure and stimulation protocol. **(a)** Design for the randomized, single-blind, and sham-controlled study. **(b)** Electrode montage with two 2 × 1 tACS arrays. Red dots are the current given positive, blue dots are the current given negative, and specific current parameters are marked on the graph. **(c)** The waveforms of electrical stimulation applied in AM-tACS, tACS and Sham groups, respectively.

In this single-blind, sham-controlled, parallel-group study, each participant attended in the PRACTICE session of the day before the experiment and in the three major sessions of the experiment: PRE, During, and POST stimulation (Figure 1a). During the PRE session, participants completed a baseline memory task. Meanwhile, their EEG data and keyboard responses were recorded as baseline data. After completing the task, the participants took a 20-min break. While the participants were resting, the experimenters analyzed their EEG data using phase-locked value (PLV) to determine the optimal frequency of stimulation. During the Stimulation session, participants performed a second memory task, lasting about 18 min. At the same time, participants were given electrical stimulation during the memory maintenance phase. Participants in the AM-tACS group received AM-tACS with a carrier frequency of 200 Hz and a baseband signal frequency determined from their baseline EEG data. Participants in the tACS group received traditional tACS. Participants in the Sham group received a sham stimulation protocol that mimicked the active stimulation condition by including an initial 10-s ramp-up period and a subsequent 10-s ramp-down period (Figure 1c). The two brain divisions that received electrical stimulation in the tACS and AM-tACS groups were the left prefrontal cortex (LPFC) and the left temporal lobe (LTC) (Figure 1b). After completing the task, the participants took a 3-min break. During the POST session, the participants performed a third memory task, and their EEG data and keyboard responses were recorded to compare performance before and after the stimulus.

**FIGURE 2:**
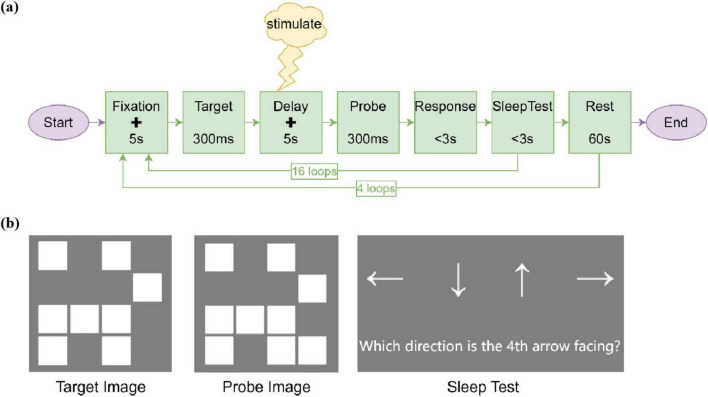
Memory task. **(a)** The design of memory task, including the whole process of the task and the presentation time and number of cycles of the stimulus. The plus sign (+) represents a fixation cross displayed at the center of the screen throughout the fixation and delay periods. During the experimental stimulus period, the stimulus was given at the delay of the memory task. **(b)** Examples of stimuli in memory tasks. The Target Image was randomly filled with 8 white squares. The probe image had a 50% chance of being identical to the target image. Alternatively, it could have one additional white square compared to the Target Image. During the drowsiness detection, a randomly sequenced series of up, down, left, and right arrows were displayed on the screen, followed by a prompt asking participants to indicate the direction of a specific arrow using the corresponding arrow keys on the keyboard.

Subjects performed memory tasks on a computer for about 18 min at a time. Each trial involved the participants memorizing a Target image and maintaining their memory for 5 s. Subsequently, participants had 3 s to indicate whether the Probe image matched the Target image by pressing the “1” key for match or the “0” key for non-match. Notably, during the 5-s delay period (i.e., the memory maintenance phase following the 300-ms Target image offset), participants in the AM-tACS group received amplitude-modulated tACS, those in the tACS group received conventional tACS, while those in the Sham group received sham stimulation (Figure 2a). The Target images consisted of randomly generated square patterns, with task difficulty parametrically controlled by varying the number of squares (Figure 2b).This approach ensured novel stimulus generation for each trial to minimize long-term memory effects while maintaining standardized administration across participants. To sustain vigilance during extended sessions, the paradigm incorporated 60-s rest periods between blocks and a drowsiness detection task requiring arrow direction discrimination using keyboard arrow keys (3-s response window).

To ensure that participants can accurately complete the memory task in the formal experiment, a practice session will be conducted 1 day before the experiment. The primary purpose of practice is to familiarize participants with the memory task and response techniques. Participants are required to practice at least three sets of memory task paradigms, achieving a correct response rate of 60% or higher with a stable trend and no further upward trend. This aims to minimize the impact of increased proficiency during the formal experiment. Brain signals will not be recorded during the practice process.

### 2.3 EEG data acquisition and preprocessing

NeuroScan SynAmps RT 64-channel Amplifier was used to record EEG data, and an additional 32-channel cap was used in some subjects. For all recordings, the amplifier was set to DC-mode with a dynamic range of ±430 mV, a resolution of 51 nV/bit, and a range of 24 bit. EEG was recorded at 1000 Hz with an anti-aliasing filter applied at 125 Hz. Electrode impedances were maintained below 10 kΩ or excluded from the analysis.

Electroencephalography data preprocessing was conducted using the EEGLAB toolbox (v2019.1) implemented in MATLAB R2013a platform. The raw data were initially downsampled to 1000 Hz to enhance computational efficiency, followed by application of a 40 Hz low-pass filter to eliminate high-frequency noise components. For resting-state data analysis, we performed 1 Hz high-pass filtering to remove DC drift artifacts, with subsequent re-referencing to the average reference and segmentation into 2000 ms epochs. Task-related data processing involved 0.1 Hz high-pass filtering combined with notch filtering (48–52 Hz) to mitigate power line interference, using bilateral mastoid (T7/T8) as the reference. Epochs were extracted from −200 ms to +800 ms relative to stimulus onset for event-related analysis. Rigorous quality control measures were implemented, including visual inspection for artifact rejection, spherical interpolation for bad channel replacement, and independent component analysis (ICA) to identify and remove physiological artifacts such as ocular movements and muscle activity. This standardized preprocessing pipeline ensured the reliability of subsequent analytical procedures.

### 2.4 Stimulation protocol

#### 2.4.1 Individualized frequency selection based on phase synchronization

The individualized frequency selection protocol was specifically developed for amplitude-modulated transcranial alternating current stimulation (AM-tACS) parameters, derived from electrophysiological markers of functional connectivity, guided by established principles of neural entrainment and synaptic plasticity. Phase-Locking Value (PLV) analysis was employed to quantify phase coherence between the left prefrontal cortex (LPFC, F3 electrode) and left temporal cortex (LTC, T7 electrode) during the memory maintenance period (500–3500 ms post-stimulus). PLV was calculated as:


$$P\,L\,V=\left|\frac{1}{N}\,\sum_{t=1}^{N}e^{i\,\left(\phi_{1}\,\left(t\right)-\phi_{2}\,\left(t\right)\right)}\right|$$

where *N* represents the number of time points, and *ϕ*_1_ (*t*) and *ϕ*_2_ (*t*) denote the instantaneous phase angles of signals from F3 and T7, respectively.

Spectral decomposition was performed across the theta frequency band (4–8 Hz) with 0.1 Hz resolution using single-trial EEG data from the baseline session. The frequency demonstrating maximal PLV for each participant was selected as their individualized baseband modulation frequency for AM-tACS, subsequently rounded to the nearest 0.5 Hz increment (e.g., 7.2 Hz → 7.0 Hz; 7.3 Hz → 7.5 Hz) to accommodate device limitations. This approach aligns with dynamic systems theory, wherein neural modulation is optimized when external stimulation frequency approximates the endogenous resonant frequency of the targeted network (Ali et al., 2013).

#### 2.4.2 Stimulation parameters and waveform configuration

Stimulation was delivered using an M × N nine-channel high-definition transcranial current stimulator (Soterix Medical) with two distinct protocols:

##### 2.4.2.1 Amplitude-modulated tACS (aM-tACS)

The waveform was generated by modulating a 200 Hz carrier frequency with the individualized theta rhythm:


$$s\,\left(t\right)=\frac{1}{2}\,\left[1+\cos\,\left(2\,\pi \,f_{m}\,t\right)\right]\,\cos\,\left(2\,\pi \,f_{c}\,t\right)$$

where *f_c_* = 200 Hz (carrier frequency) and *f_m_* represents the participant-specific theta frequency (4–8 Hz). This configuration combines the depth penetration advantages of high-frequency stimulation with the frequency-specific effects of theta entrainment (Witkowski et al., 2016; Negahbani et al., 2018; Haslacher et al., 2023).

##### 2.4.2.2 Conventional tACS

A pure sinusoidal waveform was administered at the individualized theta frequency without carrier modulation.

All stimulations were delivered at 1.5 mA peak-to-peak intensity through electrode arrays positioned over LPFC and LTC regions, with real-time impedance maintained below 10 kΩ. The sham condition replicated active stimulation’s physical sensations through brief ramp-up/ramp-down periods without sustained current delivery.

### 2.5 Statistical analysis

For statistical analysis, this study utilized Python 3.8.16 as the software tool. The dependent packages and their respective versions are listed in Table 1.

**TABLE 1:** Python dependencies for statistical analysis.

Name	Version	Description
Matplotlib	3.7.3	Basic plotting library.
Numpy	1.24.4	Matrix computation library.
Pandas	2.0.3	2D data table analysis toolkit.
Scipy	1.8.1	Scientific computing library with basic statistical analysis tools.
Seaborn	0.13.1	Advanced plotting library.
Statsmodels	0.13.5	Powerful statistical analysis library.

Behavioral measures encompassing accuracy, sensitivity, specificity, d’, and reaction time (RT) were analyzed using parametric tests after verifying their assumptions. One-way Analysis of Variance (ANOVA) was employed to examine differences in behavioral indicators across three time points (before, during, and after stimulation) within each group, as well as improvement differences between groups during and after stimulation. For reaction time data, which typically exhibits right-skewed distribution, logarithmic transformation was applied prior to parametric analysis, with non-parametric Kruskal-Wallis tests conducted as robustness checks. All multiple comparisons were adjusted using false discovery rate (FDR) correction with *q* < 0.05, and effect sizes are reported as partial eta-squared (η^2^) for ANOVA and Cohen’s d for *t*-tests to facilitate effect magnitude interpretation.

Phase locking value (PLV) analysis focused on the theta frequency band (4–8 Hz) based on established literature linking theta oscillations to working memory processes. PLV changes between frontal-occipital regions and F3-T7 electrode pairs were examined using paired sample *t*-tests to compare pre- versus post-stimulation differences within each group, with FDR correction applied for multiple frequency band and channel comparisons. Between-group differences in post-stimulation PLV changes were evaluated using one-way ANOVA for frontal-occipital comparisons and independent sample *t*-tests for F3-T7 comparisons between sham and tACS groups. The significance threshold was set at α = 0.05 for primary analyses, with appropriate adjustments made for multiple comparisons throughout all statistical tests.

## 3 Results

### 3.1 Statistics of participants

The results of the demographic analysis of the three subject groups are presented in Table 2.

**TABLE 2:** Statistics of participants.

Variables	Sham	tACS	AM-tACS	F	p
N	10	12	11	2.301	0.316
Gender (male, %)	6 (60.0%)	5 (41.6%)	3 (27.3%)		
Age (years)	22.0 ± 2.6	22.2 ± 3.1	22.7 ± 3.8	0.139	0.871
Education (years)	15.6 ± 2.9	15.6 ± 2.2	16.5 ± 3.1	0.443	0.646

### 3.2 Analysis of behavior

**FIGURE 3:**
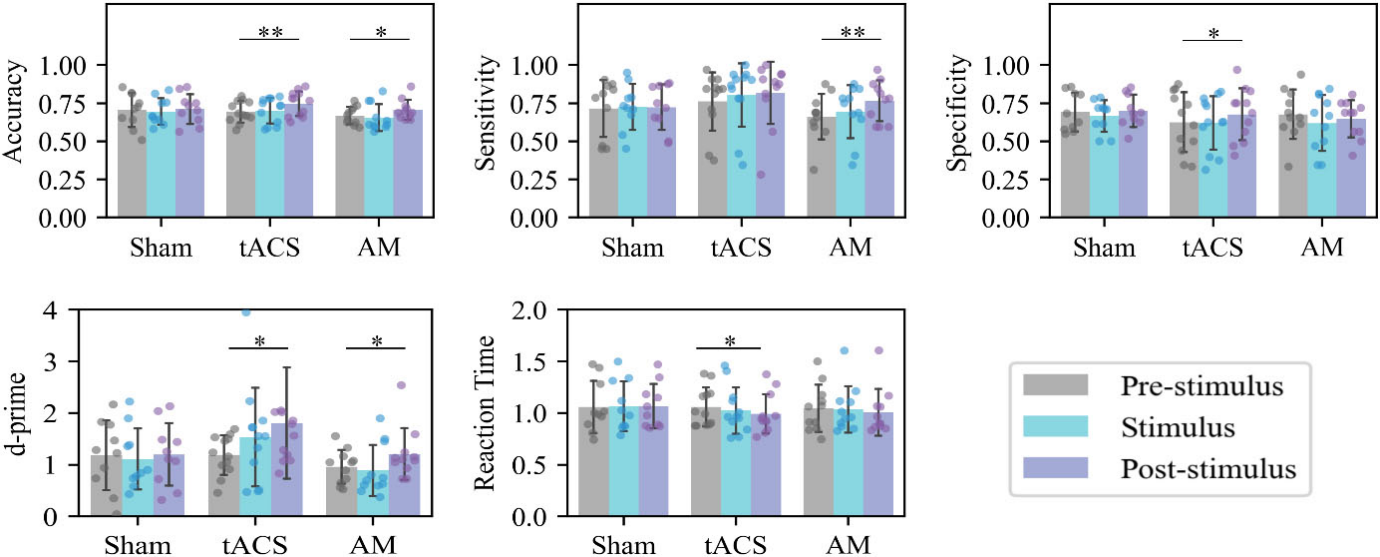
Intragroup analysis of behavior. *p < 0.05, **p < 0.01. Statistical analysis of the behavioral data at different sessions of the same group.

Figure 3 shows the accuracy, sensitivity, specificity, d’ and RT of memory tasks in the Pre-stimulus, Stimulus, and Post-stimulus session of the AM-tACS, tACS, and Sham groups. In the Sham group, there were no statistically significant differences between Pre-stimulus, Stimulus, and Post-stimulus. In the tACS group, Post-stimulus compared to Pre-stimulus, there were significant increases in accuracy (*p* = 0.002), specificity (*p* = 0.036), and d’ (*p* = 0.039), a significant decrease in reaction time (*p* = 0.024), and a slight increase in sensitivity (*p* = 0.086) that was not statistically significant. In the AM-tACS group, Post-stimulus compared to Pre-stimulus, there were significant increases in accuracy (*p* = 0.023), sensitivity (*p* = 0.008), and d’ (*p* = 0.040), while specificity (*p* = 0.363) showed a slight decrease that was not statistically significant.

**FIGURE 4:**
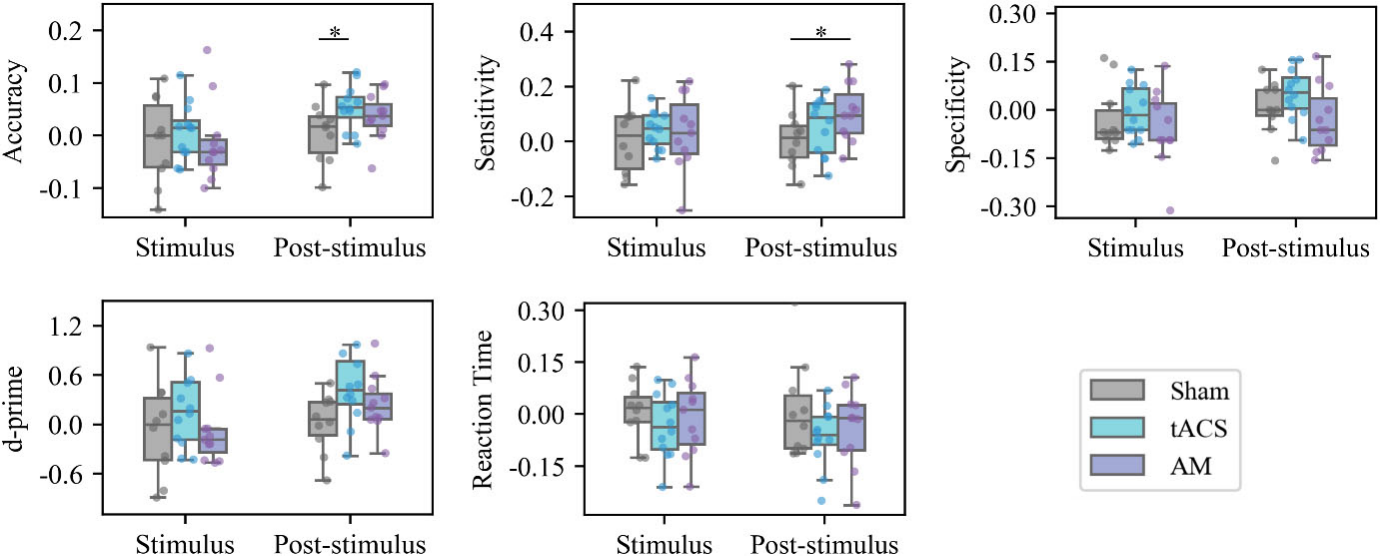
Intergroup analysis of behavior. *p < 0.05. Statistical analysis of the behavioral data of different groups in the same session. All values in the figure represent the value of the Stimulus task or the Post-Stimulus task minus the value of the Pre-Stimulus task.

As shown in Figure 4, there were no statistically significant differences in the magnitude of improvement from baseline to stimulation between any of the three groups. In terms of the magnitude of task improvement after stimulation compared to baseline, the tACS group showed a greater improvement in accuracy than the Sham group (*p* = 0.041) with a statistically significant difference. The AM-tACS group showed a greater improvement in sensitivity than the Sham group (*p* = 0.046) with a statistically significant difference. The AM-tACS group showed a smaller improvement in specificity than the tACS group (*p* = 0.051) but the difference was not statistically significant. The AM-tACS group showed a higher trend of improvement in accuracy compared to the Sham group (*p* = 0.196) and a lower trend of improvement compared to the tACS group (*p* = 0.379), but neither difference was statistically significant.

As shown in Figure 4, there were no statistically significant differences in the altered values of accuracy, sensitivity, specificity, d’, and RT of the three groups at the time of Stimulus (compared with Pre-stimulus). As for the change of behavioral indicators during stimulation, the accuracy improvement in tACS group was greater than that in Sham group (*p* = 0.041), and the sensitivity improvement in AM-tACS group was greater than that in Sham group (*p* = 0.046), and it was statistically significant. In addition, the specificity changes in the AM-tACS group were smaller than those in the tACS group (*p* = 0.051), the AM-tACS group had a tendency to increase d’ compared with the Sham group (*p* = 0.196), and had a tendency to decrease RT compared with the Sham group (*p* = 0.379), but the differences were not statistically significant.

### 3.3 EEG analysis

The EEGLAB toolbox in Matlab software was used for EEG data analysis, the reference electrodes were set as M1 and M2, the fragments with bad guide and poor signal quality were removed, and the artifact components such as eye movement and electrocardio were selected and eliminated combined with independent component analysis methods.

#### 3.3.1 PLV in the whole brain

The recorded EEG included 64 channels and 32 channels. For the purpose of uniform analysis within the group, the same 28 electrodes were retained and PLV analysis was performed after the electrode positions of the two EEG electrodes were mapped one by one. Firstly, the PLV between 28 channel electrodes in the range of 6–8 Hz was calculated for the pre-processed EEG data, and the connection matrix obtained was shown in Supplementary Figure 1.

Phase-locking value connection matrix heat maps of Sham, tACS and AM-tACS groups at baseline and after stimulation showed that the connections within the frontal lobe, between the frontal lobe and the central region, and between the parietal lobe and the central region were significantly stronger than those between other brain regions. The change of PLV in each group from baseline to post-stimulus was not obvious from the connection matrix heat map (Supplementary Figure 1a). By subtracting the baseline PLV from the PLV after stimulation, the connection matrix heat maps for the three groups were drawn (Supplementary Figure 1b). Compared with the other two groups, a greater number of PLV values were found to decrease in the AM-tACS group, but there was a certain pattern in the channels of PLV increase, which were concentrated between the channels in the frontal and occipital regions. This means that AM stimulation may promote the integration of visual information and cognitive functions by enhancing functional connectivity between frontal and occipital regions, thereby improving performance on memory tasks.

**FIGURE 5:**
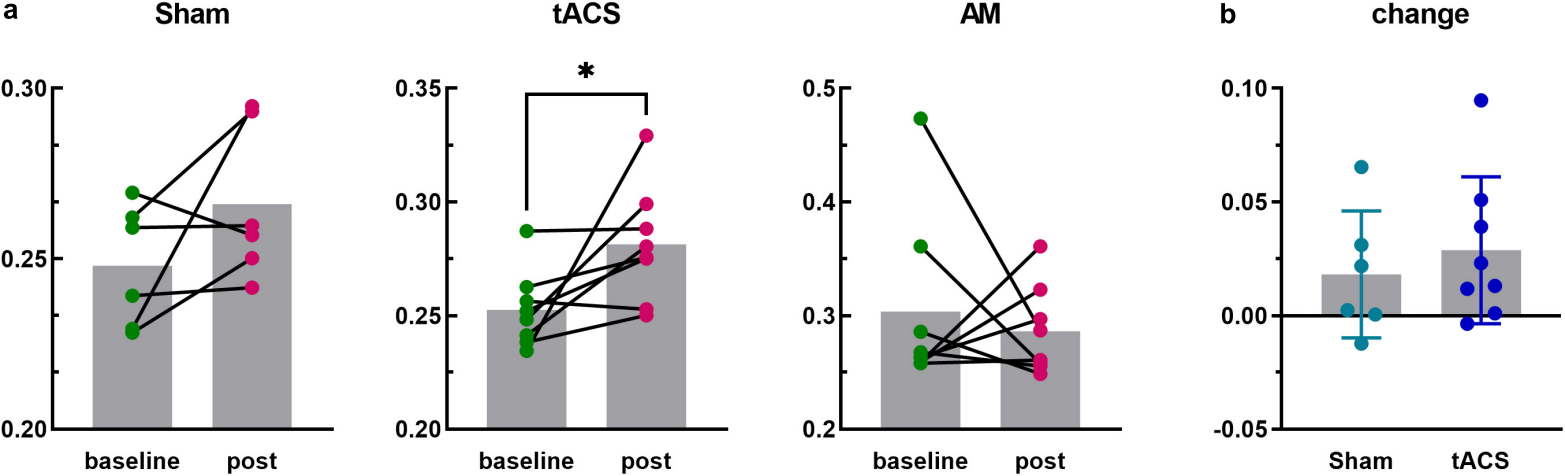
The PLV analysis of channels F3 and T7. *p < 0.05. **(a)** PLV values at baseline and post-stimulus in the Sham, tACS and AM-tACS groups. **(b)** PLV changes from baseline to post-stimulus in the Sham and tACS groups.

BrainNet Viewer is a visualization software in the field of neuroscience, which is the Matlab toolbox for network brain map visualization. Figure 5 shows the functional connection network brain map at baseline and after stimulation in the three groups of Sham, tACS and AM. From the brain network map, there was strong connectivity within the frontal, between frontal and central regions, between central and parietal, parietal, occipital, temporal, and temporal and parietal regions during memory tasks. During the post-stimulus task, interchannel connectivity within both frontal and parietal regions was higher in the tACS than Sham group than the AM-tACS group, but the connectivity between the frontal and occipital regions was higher in the AM-tACS group than in the tACS group.

Both Supplementary Figures 1, 2 showed similar functional connectivity during the task, with the strongest connectivity within the frontal, frontal and central, and the occipital lobe, followed by the internal temporal, parietal, parietal and central regions, and again between the frontal and occipital regions. Except for connections within the temporal lobe, more connected connections are concentrated in the midline and sides of the brain. Taking into account the overall change from baseline to post-stimulus, PLV values were enhanced in the Sham and tACS groups, and slightly decreased in the AM-tACS group. However, frontal occipital PLV was significantly increased in AM-tACS group after stimulation.

#### 3.3.2 PLV between frontal and occipital lobes

**FIGURE 6:**
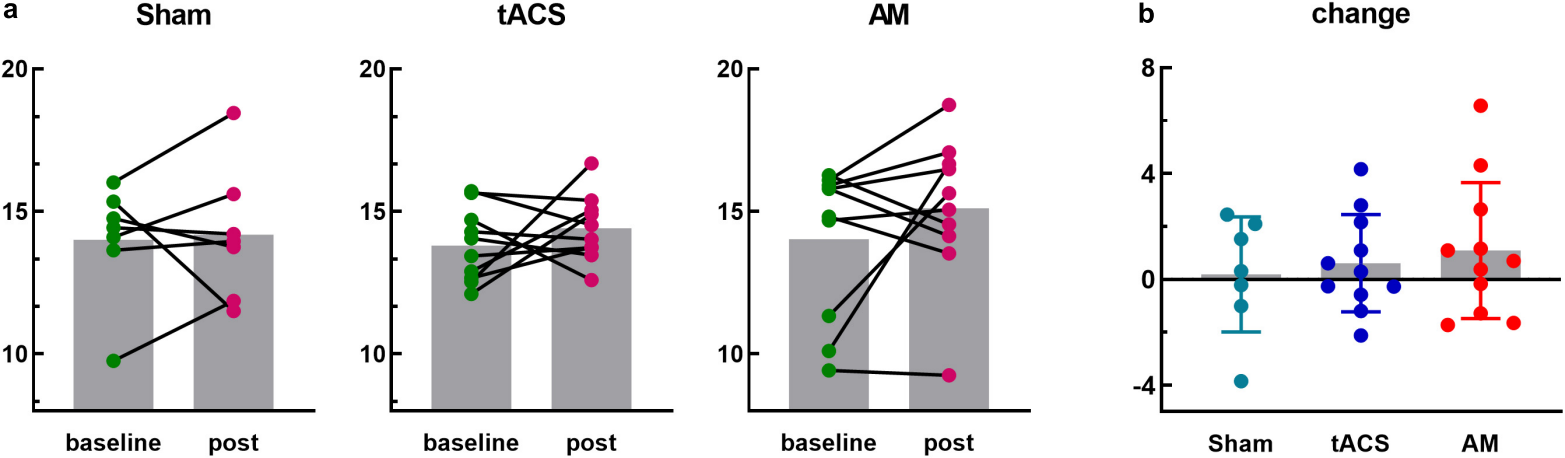
Phase-locking value (PLV) in the frontal and occipital lobes. **(a)** An independent-samples *t*-test was conducted to compare the total PLV between 10 frontal channels and 3 occipital channels at baseline and post-stimulus sessions in the Sham, tACS, and AM-tACS groups. **(b)** A one-way ANOVA was performed to analyze the changes in PLV from baseline to post-stimulus in the Sham, tACS, and AM-tACS groups.

The sum of PLV values between frontal lobe (including 10 channels FP1, FP2, F7, F3, FZ, F4, F8, FC3, FCZ, FC4) and occipital lobe (including 3 channels O1, OZ, O2) were further analyzed. The group average of PLV in fronto-occipital lobe after stimulation was improved in all three groups, but there was no significant difference (Sham: *t* = 0.225, *p* = 0.830; tACS: *t* = 0.988, *p* = 0.349; AM: *t* = 1.271, *p* = 0.236). In addition, the degree of improvement in the AM-tACS group was greater than that in the tACS group than that in the Sham group (Figure 6a). However, one-way ANOVA was used to analyze the change value of fronto-occipital PLV, and the results were not statistically significant (*F* = 0.366, *p* = 0.697) (Figure 6b).

#### 3.3.3 PLV between F3 and T7 channels

In this study, the central electrodes of the two brain regions stimulated by electrical stimulation were F3 and T7, respectively. In order to explore the influence of neuromodulation on the functional connectivity of the target interval, the PLV values between F3 and T7 electrodes in each group were further analyzed. Figure 5 shows the PLV values of the F3 and T7 channels for all subjects at baseline and during the post-stimulus task, and the results are generally consistent with the overall changes in all channels, with PLV increasing in the Sham (*t* = 1.591, *p* = 0.172) and tACS (*t* = 2.515, *p* = 0.040) groups and decreasing in the AM-tACS group (*W* = −6.000, *p* = 0.742). Although PLV values were significantly improved in the tACS group after stimulation, the degree of improvement was not statistically significant compared to the Sham group (*t* = 0.6423, *p* = 0.5328).

## 4 Discussion

### 4.1 Modulatory effects of AM-tACS on working memory

The differential effects of AM-tACS compared to conventional tACS reveal important insights into the neural mechanisms of working memory enhancement. Our findings demonstrate that while both stimulation approaches improved working memory performance relative to sham, they operated through distinct temporal patterns and neural pathways. Conventional tACS produced immediate performance benefits during stimulation, consistent with its well-documented ability to entrain local gamma oscillations in targeted cortical regions (Manippa et al., 2024; Rosato et al., 2025). In contrast, AM-tACS exhibited delayed but more sustained effects emerging post-stimulation, suggesting its primary action involves network-level reorganization rather than local entrainment (Omae et al., 2024).

The delayed emergence of AM-tACS effects aligns with the phase-resetting hypothesis of rhythmic stimulation, where external oscillations gradually synchronize distributed neural populations by resetting ongoing activity patterns. Specifically, the 200 Hz carrier frequency, amplitude-modulated by an individualized theta-range signal, appears to enhance working memory through coordinated theta-gamma coupling between prefrontal and parietal regions. This cross-frequency interaction likely facilitates phase-amplitude coupling, where the endogenously derived (4–8 Hz) theta rhythms modulate the amplitude of high-frequency (∼200 Hz) gamma oscillations, creating optimal temporal windows for information transfer between executive and storage networks (Papaioannou et al., 2025). This mechanism differs fundamentally from conventional tACS, which primarily induces local gamma entrainment at a single frequency without the sophisticated cross-frequency coordination characteristic of AM-tACS.

The behavioral profile of AM-tACS effects provides further support for this network-level account. The selective enhancement of sensitivity measures, peaking 15–20 min post-stimulation, specifically implicates modulation of the central executive’s monitoring functions in Baddeley’s multicomponent model, rather than the phonological or visuospatial storage subsystems (Poole et al., 2021). This temporal and functional specificity suggests that AM-tACS may preferentially optimize the dynamic interactions between prefrontal control systems and posterior storage regions, rather than enhancing local processing within either region alone. The transient performance decrements observed during active stimulation may reflect initial phase competition between the exogenous 6 Hz modulation and endogenous theta rhythms, with the subsequent behavioral rebound indicating successful network reorganization once this competition resolves.

### 4.2 Modulatory effects of AM-tACS on brain activity

The differential effects of AM-tACS on functional connectivity patterns provide compelling evidence for its network-modulating properties. Phase locking value analysis revealed that while conventional tACS enhanced local synchronization specifically between the stimulated sites (F3-T7), AM-tACS showed a distinct tendency to strengthen long-range frontoparietal connectivity. This spatial specificity mirrors the known frequency dependencies of different neural pathways-with frontoparietal interactions particularly reliant on theta-gamma coupling, while fronto-occipital communication depends more on alpha-band synchronization (Jones et al., 2020).

The dual-frequency nature of AM-tACS appears critical for its ability to simultaneously maintain local excitatory-inhibitory balance while coordinating activity across distant regions. The 200 Hz carrier frequency provides local entrainment of high-frequency gamma oscillations, while the individualized theta-range amplitude modulation appears to reset the phase of endogenous theta rhythms coordinating interactions between regions. Computational modeling suggests this combination is particularly effective at 15% modulation depth, which optimally balances local and network-level effects (Hosseinian et al., 2021; Karimi et al., 2024). Weaker modulation fails to drive sufficient cross-regional coupling, while stronger modulation disrupts the stability of local oscillations.

This “frequency-space coupling” framework helps explain several key observations. First, it accounts for why AM-tACS shows superior effects on distributed network interactions compared to conventional tACS. Second, it explains the individual variability in response patterns, as the effectiveness of cross-frequency coupling depends on baseline connectivity states (Vakorin et al., 2021). Finally, it provides a mechanistic basis for the behavioral dissociation between immediate local effects (conventional tACS) and delayed network-level enhancements (AM-tACS) (Fiene et al., 2020). The correlation between frontoparietal PLV increases and working memory improvements further supports the behavioral relevance of these neurophysiological changes.

These findings collectively suggest that AM-tACS operates through a unique combination of local entrainment and network-level coordination, distinguishing it from conventional tACS approaches. The delayed emergence of behavioral benefits, coupled with the specific enhancement of distributed connectivity patterns, points to AM-tACS’s potential for targeting network-level dysfunctions in cognitive disorders. Future research should explore whether these network-modulating properties can be optimized through personalized parameter selection based on individual connectivity profiles.

### 4.3 Limitations

Several methodological constraints warrant consideration. First, the modest sample sizes (Sham: *n* = 10; tACS: *n* = 12; AM-tACS: *n* = 11) may limit statistical power, particularly for detecting subtle network-level effects. Second, while our task design controlled for several confounding factors, the absence of concurrent EEG recording during stimulation prevents direct observation of neural entrainment dynamics. Third, while we employed individualized modulation frequencies based on pre-task EEG, the fixed 200 Hz carrier frequency represents only one point in a large parameter space that future studies should systematically explore, including variations in both carrier frequency and modulation parameters.

### 4.4 Safety of AM-tACS

The safety profile of AM-tACS in this study matched conventional tACS, with participants reporting only mild transient sensations (tingling, pulsation). No severe adverse effects or lasting sequelae were observed during 30-days follow-up. These findings support the continued investigation of AM-tACS as a well-tolerated neuromodulation approach, though larger safety studies remain warranted.

## 5 Conclusion

Collectively, our study provides preliminary evidence that AM-tACS may effectively modulate working memory performance in college students, with observed behavioral effects exceeding the field’s average reported effect size (*d* = 0.07) for conventional tACS. While the modulation patterns of AM-tACS appear comparable to traditional tACS in our data–suggesting its potential as an alternative for closed-loop neuromodulation systems–these conclusions must be tempered by the study’s limitations. Given the modest sample size (Sham = 10, tACS = 12, AM-tACS = 11) and inherent interindividual variability in tACS responsiveness, future replication in larger cohorts (*N* > 80 per group) with rigorous controls is essential to validate these findings. We recommend that subsequent research: (1) incorporate individualized stimulation parameters (e.g., frequency adaptation based on resting-state EEG), (2) employ multimodal neuroimaging (e.g., concurrent tACS-fMRI) to elucidate mechanism-specific effects, and (3) systematically compare AM-tACS with conventional tACS in closed-loop paradigms to assess their relative clinical utility.

## Data Availability

The raw data supporting the conclusions of this article will be made available by the authors, without undue reservation.

## Supplementary material

Supplementary Figure 1 Phase-locking value (PLV) connection matrix heat map. **(a)** Heatmap of the baseline and post-stimulus PLV connectivity matrix for the Sham, tACS and AM-tACS groups. **(b)** PLV change values for the three groups, Sham, tACS, and AM, were subtracted from baseline after stimulation.

Supplementary Figure 2 Network brain map of PLV functional connectivity. The node is the channel, the node color shows the brain partition, red is the frontal area, blue is the temporal area, green is the central area, purple is the parietal area, yellow is the occipital area. Edges represent the PLV between different channels, the thicker the edges represent the closer the connections, the connections between the same brain region correspond to the color of the nodes, the gray edges are the connections between different brain regions, and the display threshold of the edge is set to 0.2. **(a)** Brain maps of the mean PLV functional connectivity network in the lateral, medial, and dorsal regions across all subjects during the baseline WM task. **(b)** PLV functional connection network brain maps for Sham, tACS and AM-tACS groups in the sagittal direction of the single view during post-stimulus WM task.

## References

[B1] Ali M. Sellers K. Fröhlich F. (2013). Transcranial alternating current stimulation modulates large-scale cortical network activity by network resonance. *J. Neurosci.* 33 11262–11275. 10.1523/JNEUROSCI.5867-12.2013 23825429 PMC6618612

[B2] Asamoah B. Khatoun A. Mc Laughlin M. (2022). Frequency-specific modulation of slow-wave neural oscillations via weak exogeneous extracellular fields reveals a resonance pattern. *J. Neurosci.* 42 6221–6231. 10.1523/JNEUROSCI.0177-22.2022 35790404 PMC9374140

[B3] Baddeley A. (2012). Working memory: Theories, models, and controversies. *Annu. Rev. Psychol.* 63 1–29. 10.1146/annurev-psych-120710-100422 21961947

[B4] Benussi A. Cantoni V. Grassi M. Brechet L. Michel C. Datta A. (2022). Increasing brain gamma activity improves episodic memory and restores cholinergic dysfunction in Alzheimer’s disease. *Ann. Neurol.* 92 322–334. 10.1002/ana.26411 35607946 PMC9546168

[B5] Booth S. Taylor J. Brown L. Pobric G. (2022). The effects of transcranial alternating current stimulation on memory performance in healthy adults: A systematic review. *Cortex* 147 112–139. 10.1016/j.cortex.2021.12.001 35032750

[B6] Borghini G. Candini M. Filannino C. Hussain M. Walsh V. Romei V. (2018). Alpha oscillations are causally linked to inhibitory abilities in ageing. *J. Neurosci.* 38 4418–4429. 10.1523/JNEUROSCI.1285-17.2018 29615485 PMC6596011

[B7] Dunning D. Westgate B. Adlam A. R. (2016). A meta-analysis of working memory impairments in survivors of moderate-to-severe traumatic brain injury. *Neuropsychology* 30 811–819. 10.1037/neu0000285 27182710

[B8] Fiene M. Schwab B. Misselhorn J. Herrmann C. Schneider T. Engel A. (2020). Phase-specific manipulation of rhythmic brain activity by transcranial alternating current stimulation. *Brain Stimul.* 13 1254–1262. 10.1016/j.brs.2020.06.008 32534253

[B9] Grot S. Légaré V. Lipp O. Soulières I. Dolcos F. Luck D. (2017). Abnormal prefrontal and parietal activity linked to deficient active binding in working memory in schizophrenia. *Schizophr. Res.* 188 68–74. 10.1016/j.schres.2017.01.021 28095997

[B10] Grover S. Wen W. Viswanathan V. Gill C. Reinhart R. (2022). Long-lasting, dissociable improvements in working memory and long-term memory in older adults with repetitive neuromodulation. *Nat. Neurosci.* 25 1237–1246. 10.1038/s41593-022-01132-3 35995877 PMC10068908

[B11] Haslacher D. Narang A. Sokoliuk R. Cavallo A. Reber P. Nasr K. (2023). In vivo phase-dependent enhancement and suppression of human brain oscillations by transcranial alternating current stimulation (tACS). *Neuroimage* 275:120187. 10.1016/j.neuroimage.2023.120187 37230205

[B12] Haslacher D. Nasr K. Robinson S. Braun C. Soekadar S. (2021). Stimulation artifact source separation (SASS) for assessing electric brain oscillations during transcranial alternating current stimulation (tACS). *Neuroimage* 228:117571. 10.1016/j.neuroimage.2020.117571 33412281 PMC7903161

[B13] Hosseinian T. Yavari F. Kuo M. Nitsche M. Jamil A. (2021). Phase synchronized 6 Hz transcranial electric and magnetic stimulation boosts frontal theta activity and enhances working memory. *Neuroimage* 245:118772. 10.1016/j.neuroimage.2021.118772 34861393

[B14] Hsu C. Liu T. Lee D. Yeh D. Chen Y. Liang W. (2023). Amplitude modulating frequency overrides carrier frequency in tACS-induced phosphene percept. *Hum. Brain Mapp.* 44 914–926. 10.1002/hbm.26111 36250439 PMC9875935

[B15] Johnson E. Lin J. King-Stephens D. Weber P. Laxer K. Saez I. (2023). A rapid theta network mechanism for flexible information encoding. *Nat. Commun.* 14:2872. 10.1038/s41467-023-38574-7 37208373 PMC10198978

[B16] Jones K. Johnson E. Berryhill M. (2020). Frontoparietal theta-gamma interactions track working memory enhancement with training and tDCS. *Neuroimage* 211:116615. 10.1016/j.neuroimage.2020.116615 32044440 PMC7733399

[B17] Karimi N. Amirfattahi R. Zeidaabadi Nezhad A. (2024). Neuromodulation effect of temporal interference stimulation based on network computational model. *Front. Hum. Neurosci.* 18:1436205. 10.3389/fnhum.2024.1436205 39386280 PMC11461302

[B18] Kasten F. Negahbani E. Fröhlich F. Herrmann C. (2018). Non-linear transfer characteristics of stimulation and recording hardware account for spurious low-frequency artifacts during amplitude modulated transcranial alternating current stimulation (AM-tACS). *Neuroimage* 179 134–143. 10.1016/j.neuroimage.2018.05.068 29860086

[B19] Le T. Borghi J. Kujawa A. Klein D. Leung H. (2017). Alterations in visual cortical activation and connectivity with prefrontal cortex during working memory updating in major depressive disorder. *Neuroimage Clin.* 14 43–53. 10.1016/j.nicl.2017.01.004 28138426 PMC5257188

[B20] Maehler C. Schuchardt K. (2016). Working memory in children with specific learning disorders and/or attention deficits. *Learn. Individ. Dif.* 49 341–347. 10.1016/j.lindif.2016.05.007 18625783

[B21] Manippa V. Palmisano A. Nitsche M. Filardi M. Vilella D. Logroscino G. (2024). Cognitive and neuropathophysiological outcomes of Gamma-tACS in dementia: A systematic review. *Neuropsychol. Rev.* 34 338–361. 10.1007/s11065-023-09589-0 36877327 PMC10920470

[B22] Negahbani E. Kasten F. Herrmann C. Fröhlich F. (2018). Targeting alpha-band oscillations in a cortical model with amplitude-modulated high-frequency transcranial electric stimulation. *Neuroimage* 173 3–12. 10.1016/j.neuroimage.2018.02.005 29427848 PMC5911251

[B23] Nissim N. Pham D. Poddar T. Blutt E. Hamilton R. (2023). The impact of gamma transcranial alternating current stimulation (tACS) on cognitive and memory processes in patients with mild cognitive impairment or Alzheimer’s disease: A literature review. *Brain Stimul.* 16 748–755. 10.1016/j.brs.2023.04.001 37028756 PMC10862495

[B24] Omae E. Shima A. Tanaka K. Yamada M. Cao Y. Nakamura T. (2024). Case report: An N-of-1 study using amplitude modulated transcranial alternating current stimulation between Broca’s area and the right homotopic area to improve post-stroke aphasia with increased inter-regional synchrony. *Front. Hum. Neurosci.* 18:1297683. 10.3389/fnhum.2024.1297683 38454909 PMC10917932

[B25] Papaioannou O. Clark K. Ogbuagu N. Silverstein S. Thompson J. Erickson M. (2025). Theta-gamma phase-amplitude coupling in psychosis and healthy controls: Predicting working memory capacity across different tasks. *Neuroimage Clin.* 48:103839. 10.1016/j.nicl.2025.103839 40639284 PMC12343476

[B26] Phillips N. Parry L. Mandalis A. Lah S. (2017). [Formula: See text]Working memory outcomes following traumatic brain injury in children: A systematic review with meta-analysis. *Child Neuropsychol.* 23 26–66. 10.1080/09297049.2015.1085500 26397711

[B27] Poole B. Phillips N. Stewart E. Harris I. Lah S. (2021). Working memory in pediatric epilepsy: A systematic review and meta-analysis. *Neuropsychol. Rev.* 31 569–609. 10.1007/s11065-021-09491-7 33818735

[B28] Rosato M. Sala M. Coccaro A. Cutini S. Liotti M. (2025). Repetitive gamma-tACS improves the reaction times of healthy young adults in a visuospatial working memory task: A randomized study. *Brain Sci.* 15 343. 10.3390/brainsci15040343 40309790 PMC12026391

[B29] Stegmayer K. Usher J. Trost S. Henseler I. Tost H. Rietschel M. (2015). Disturbed cortico-amygdalar functional connectivity as pathophysiological correlate of working memory deficits in bipolar affective disorder. *Eur. Arch. Psychiatry Clin. Neurosci*. 265 303–311. 10.1007/s00406-014-0517-5 25119145

[B30] Thiele C. Zaehle T. Haghikia A. Ruhnau P. (2021). Amplitude modulated transcranial alternating current stimulation (AM-TACS) efficacy evaluation via phosphene induction. *Sci. Rep.* 11:22245. 10.1038/s41598-021-01482-1 34782626 PMC8593032

[B31] Vakorin V. Nita D. Payne E. McBain K. Frndova H. Go C. (2021). Alterations in coordinated EEG activity precede the development of seizures in comatose children. *Clin. Neurophysiol.* 132 1505–1514. 10.1016/j.clinph.2021.03.015 34023630

[B32] Wischnewski M. Alekseichuk I. Opitz A. (2023). Neurocognitive, physiological, and biophysical effects of transcranial alternating current stimulation. *Trends Cogn. Sci.* 27 189–205. 10.1016/j.tics.2022.11.013 36543610 PMC9852081

[B33] Witkowski M. Garcia-Cossio E. Chander B. Braun C. Birbaumer N. Robinson S. (2016). Mapping entrained brain oscillations during transcranial alternating current stimulation (tACS). *Neuroimage* 140 89–98. 10.1016/j.neuroimage.2015.10.024 26481671

[B34] Wu L. Liu T. Wang J. (2021). Improving the effect of transcranial alternating current stimulation (tACS): A systematic review. *Front. Hum. Neurosci.* 15:652393. 10.3389/fnhum.2021.652393 34163340 PMC8215166

[B35] Ziaei M. Salami A. Persson J. (2017). Age-related alterations in functional connectivity patterns during working memory encoding of emotional items. *Neuropsychologia* 94 1–12. 10.1016/j.neuropsychologia.2016.11.012 27865969

