# Supplementary figures and images for: The effects of amplitude modulated transcranial alternating current stimulation on working memory of college students
Source: Front Hum Neurosci. 2025 Oct 20;19:1639378. doi: 10.3389/fnhum.2025.1639378 (PMC12580318; doi:10.3389/fnhum.2025.1639378)

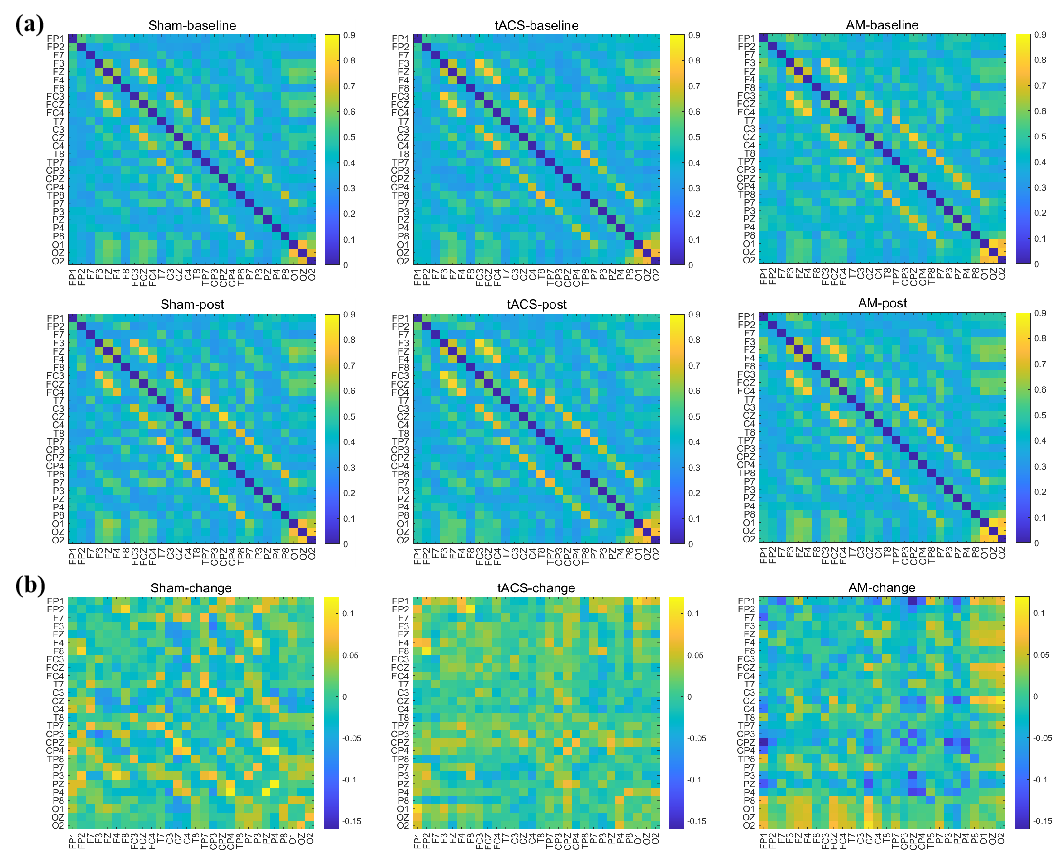

Supplement: Supplementary Figure 1 — Phase-locking value (PLV) connection matrix heat map. (a) Heatmap of the baseline and post-stimulus PLV connectivity matrix for the Sham, tACS and AM-tACS groups. (b) PLV change values for the three groups, Sham, tACS, and AM, were subtracted from baseline after stimulation. [file Image_1.tif]

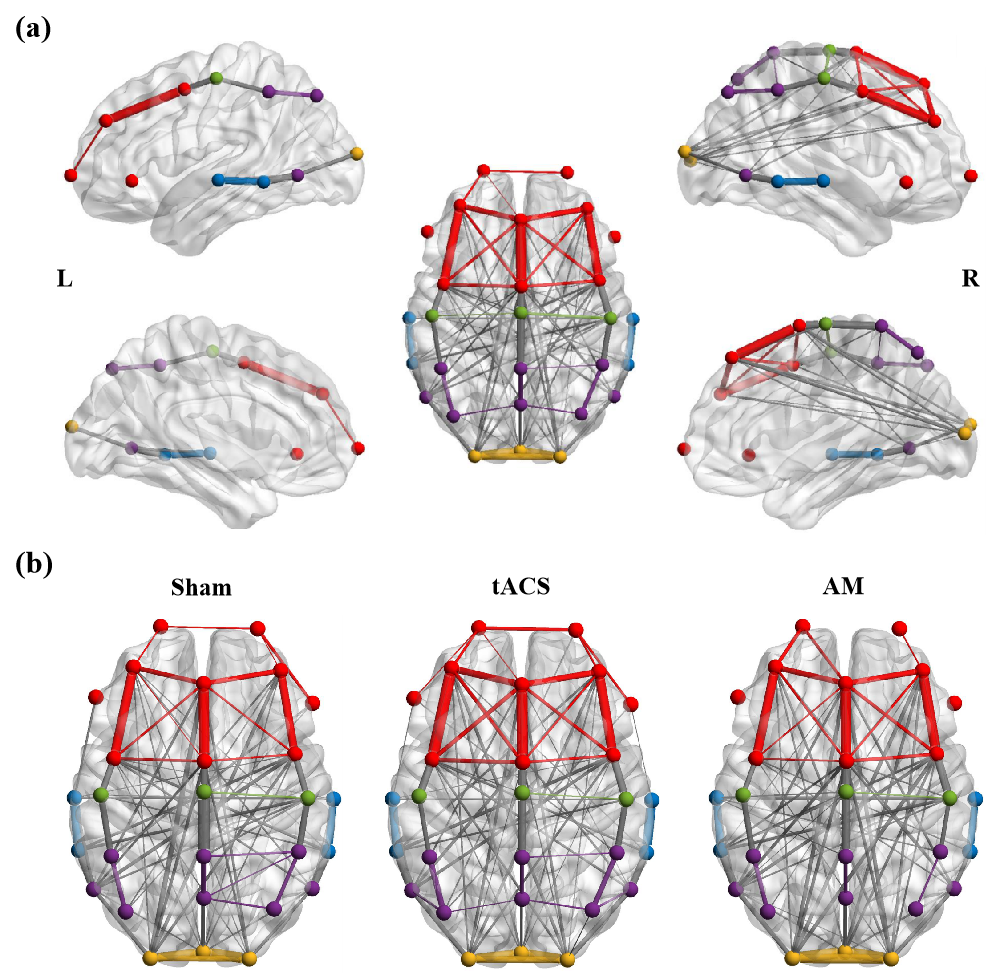

Supplement: Supplementary Figure 2 — Network brain map of PLV functional connectivity. The node is the channel, the node color shows the brain partition, red is the frontal area, blue is the temporal area, green is the central area, purple is the parietal area, yellow is the occipital area. Edges represent the PLV between different channels, the thicker the edges represent the closer the connections, the connections between the same brain region correspond to the color of the nodes, the gray edges are the connections between different brain regions, and the display threshold of the edge is set to 0.2. (a) Brain maps of the mean PLV functional connectivity network in the lateral, medial, and dorsal regions across all subjects during the baseline WM task. (b) PLV functional connection network brain maps for Sham, tACS and AM-tACS groups in the sagittal direction of the single view during post-stimulus WM task. [file Image_2.tif]
